# Exploring the efficacy and safety of Ambroxol in Gaucher disease: an overview of clinical studies

**DOI:** 10.3389/fphar.2024.1335058

**Published:** 2024-02-13

**Authors:** Feda E. Mohamed, Fatma Al-Jasmi

**Affiliations:** ^1^ Department of Genetics and Genomics, College of Medicine and Health Sciences, United Arab Emirates University, Al Ain, United Arab Emirates; ^2^ ASPIRE Precision Medicine Research Institute Abu Dhabi, United Arab Emirates University, Abu Dhabi, United Arab Emirates; ^3^ Department of Pediatrics, Tawam Hospital, Al Ain, United Arab Emirates

**Keywords:** Gaucher disease, inborn error of metabolism, sphingolipidoses, Ambroxol, pharmacological chaperones, glucocerebrosidase enzyme, enzyme enhancement therapy, drug repurposing

## Abstract

Gaucher disease (GD) is mainly caused by glucocerebrosidase (GCase) enzyme deficiency due to genetic variations in the *GBA1* gene leading to the toxic accumulation of sphingolipids in various organs, which causes symptoms such as anemia, thrombocytopenia, hepatosplenomegaly, and neurological manifestations. GD is clinically classified into the non-neuronopathic type 1, and the acute and chronic neuronopathic forms, types 2 and 3, respectively. In addition to the current approved GD medications, the repurposing of Ambroxol (ABX) has emerged as a prospective enzyme enhancement therapy option showing its potential to enhance mutated GCase activity and reduce glucosylceramide accumulation in GD-affected tissues of different *GBA1* genotypes. The variability in response to ABX varies across different variants, highlighting the diversity in patients’ therapeutic outcomes. Its oral availability and safety profile make it an attractive option, particularly for patients with neurological manifestations. Clinical trials are essential to explore further ABX’s potential as a therapeutic medication for GD to encourage pharmaceutical companies’ investment in its development. This review highlights the potential of ABX as a pharmacological chaperone therapy for GD and stresses the importance of addressing response variability in clinical studies to improve the management of this rare and complex disorder.

## 1 Introduction

### 1.1 Overview of Gaucher disease

Gaucher disease (GD, OMIM #230800) is the second most prevalent disease among lysosomal storage disorders with a worldwide incidence of about 1:40,000 to 1:60,000 births (Stirnemann et al., 2017). It is a rare autosomal recessive disorder caused by genetic variations in the *GBA1* gene leading to the expression of defective glucocerebrosidase (GCase, EC: 4.2.1.25) enzyme mainly characterized by low residual activity in affected tissues (Hruska et al., 2008). To date, there have been more than 690 different variants reported in the *GBA1* gene according to the Human Gene Mutation Database of which the majority are from the missense type (Stenson et al., 2020). Abnormal low GCase enzymatic activity consequently accumulates its glucosylceramide (GlcCer) substrate in affected organs like bone marrow, the spleen, and the liver. GlcCer accumulation may also occur in bone and neuronal tissues in different forms of the disease. Therefore, GD is subclinically categorized into types I, II, and III, considering factors such as the nature of the genetic variant, levels of residual activity, neuronal involvement, disease severity, and prognosis. Based on neuronal involvement, type I is named non-neuronopathic GD while types II and III are neuronopathic-GD (nGD) (Sidransky, 2004).

GD presents a wide range spectrum of clinical manifestations. GD type I (GD1, non-neuronopathic GD, MIM 230800) is the most common form of the disease and is primarily distinguished by the absence of neurological impairment (Linari and Castaman, 2015). The average age of onset is 10–20 years old, although milder manifestations in early childhood might be downplayed or ignored. Clinical manifestations often affect the patient’s quality of life but nowadays, when patients are usually treated, GD1-associated mortality is rare (Charrow et al., 2000). The primary clinical features of this form include splenomegaly, hepatomegaly, fatigue, gallstone formation, anemia, and thrombocytopenia. Bone involvement, seen in approximately 80% of GD1 cases due to bone marrow infiltration, can present as acute painful bone crises or chronic pain, which is assessed using tools like the Visual Analog Scale (VAS) or Numerical Rating Scale (NRS) (Wenstrup et al., 2002; Chis et al., 2021). The intensity of the pain varies, potentially influencing functional prognosis. Assessing skeletal manifestations in GD involves a combination of clinical evaluations, laboratory tests, and imaging techniques such as Magnetic Resonance Imaging (MRI) and Dual-energy X-ray absorptiometry (DEXA) (Hughes et al., 2019). While type 1 GD is typically considered the non-neuronopathic form of the disease, initial investigations into individual cases of GD1 patients have suggested a significantly higher risk of developing Parkinson’s disease (PD) (Biegstraaten et al., 2010). Bultron et al.'s study (2010) involving a cohort of 444 GD1 patients demonstrated that these individuals face an approximately 20-fold increased lifetime risk of developing PD compared to the general population (Bultron et al., 2010). Recent data further underscores a substantial correlation between *GBA1* variants (approximately 130 variants) and the onset of PD (Zhang et al., 2018; Riboldi and Di Fonzo, 2019). GD type III (GD3, also known as subacute or juvenile neuronopathic GD, MIM 231000) patients are similarly affected with the visceral symptoms mentioned in GD1 but neurological involvement has been reported before the age of 20 years in most of the cases (Stirnemann et al., 2017). Patients who are affected by this condition generally survive into their mid to early adulthood although, with treatment for the systemic manifestations some patients are now in their sixties. Systemic features include enlarged liver and spleen, anemia, thrombocytopenia, bone abnormalities, and early-onset horizontal supranuclear gaze palsy (Schwartz et al., 2017). Neurological symptoms range from cognitive impairment, saccadic eye movement abnormalities, myoclonus, seizures, dementia, ocular muscle apraxia, and muscle weakness (Schwartz et al., 2017). The disease prognosis exhibits considerable variation, as certain patients may undergo a relatively stable clinical course, whereas others may encounter more pronounced neurological manifestations (Stone et al., 2023). Clinical studies have shown that there is heterogeneity in the reported neurological impairments among patients with GD3 (Tylki-Szymańska et al., 2010). While some patients may show only mild systemic involvement, with horizontal ophthalmoplegia being their sole neurological symptom, others may experience more severe forms of the disease (Stirnemann et al., 2017). Lung involvement with interstitial lung disease has been seen in a substantial percentage of reported GD3 patients and some present with retroperitoneal lymphadenopathy that often fails to respond to the available medications (Linari and Castaman, 2015). GD patients with saposin C (GCaseactivator protein) deficiency are mostly presented with neurological manifestations comparable to that observed in the GD3 form of the disease (Mohamed et al., 2022). Technically, however, they are no longer classified as having GD as the causative mutations are in *PSAP* rather than in *GBA1* (Mohamed et al., 2022).

GD type II (GD2, known as infantile or acute neuronopathic GD, MIM 230900) is the rarest and most severe form of the disease. It typically onset at 1–6 months of age and has a mean survival age of 11.7 months (Mignot et al., 2006). In addition to visceral symptoms, neurological impairments appear initially as oculomotor abnormalities followed by brainstem involvement which may include psychomotor development delay, seizures, growth retardation, and apnea (Stirnemann et al., 2017). The fetal form of GD2 usually results in intrauterine fetal demise or early neonatal death mainly due to hydrops fetalis (Mignot et al., 2003). Overall, the clinical classification of nGD patients is complex as the symptoms may overlap between GD2 and GD3. In some instances, it is unclear if all the associated neurological symptoms are directly caused by the enzyme deficiency or if they arise as secondary effects that emerge as a consequence of the primary manifestations of the underlying GD (Sidransky, 2004). For example, neurological symptoms may aries in GD1 patients due to spinal compression fractures although type 1 GD is considered non-neuronopathic (Roshan Lal and Sidransky, 2017). Also, GD3 patients with neurological findings restricted to mild eye movement abnormalities may be misclassified as having GD1 whereas patients with genotypes usually predictive for GD3 may never have CNS manifestations. The challenge lies in distinguishing between symptoms directly attributable to the enzyme deficiency and those arising as a cascade of complications triggered by the fundamental aspects of Gaucher disease.

### 1.2 Current medicational options for GD

To date, there are two categories of clinically approved medications available for the management of GD symptoms, to improve the patient’s quality of life while also preventing irreversible damage. These categories include Enzyme Replacement Therapy (ERT) and Substrate Reduction Therapy (SRT). ERT involves the administration of recombinant enzymes to patients to replace the deficient enzyme in the body, while SRT focuses on reducing the production of the accumulated substrate that leads to GD symptoms. Despite their reported limitations and side effects, ERT and SRT are effective for ameliorating GD manifestations, and are currently considered standard treatments for this condition. ERT augments low-activity mutant GCase with a recombinant form of the normal human enzyme (Van Rossum and Holsopple, 2016). This allows the body to break down the accumulated glucocerebroside in affected organs and bone marrow. Patients receive ERT via intravenous (IV) infusion about every 2 weeks (1–2 h per session) usually taking place at an infusion center or home infusion. Imiglucerase, velaglucerase alfa, and taliglucerase alfa are the current approved medications for the treatment and management of GD within the ERT medication class (Sam et al., 2021; National Gaucher Foundation, 2023). Generally, both pediatric and adult patients with GD1 respond well to the mentioned ERT options, which significantly ameliorates the underlying clinical manifestation (Gupta and Pastores, 2018). Although it is rare, hypersensitivity reactions due to ERT drugs have been reported (Turgay Yagmur et al., 2020). Slowing the infusion rate and using the appropriate premedication will usually solve the issue, alternatively adjusting the dose, or switching to another ERT medication (Revel-Vilk et al., 2018).

SRT compounds have been introduced in the therapy of multiple lysosomal storage disorders including GD by targeting the glucocerebroside substrate synthesis pathway in the cell (Gupta and Pastores, 2018). These compounds reduce the workload on the mutated GCase by decreasing the amount of glucocerebroside for it to break down, enabling the patient’s remaining residual activity to address the accumulated substrates and prevent additional accumulation (Sam et al., 2021). SRT compounds are small molecular weight compounds that are orally administrated. Eliglustat and miglustat are currently the only clinically -approved oral SRT drugs for patients with GD (McCormack and Goa, 2003; National Gaucher Foundation, 2023). SRTs are not approved for use in individuals under 18 or women who are breastfeeding, pregnant, or trying to become pregnant, or in patients with nGD. Miglustat has significant gastrointestinal side effects while eliglustat has the potential for dose-limiting interactions with other drugs that are metabolized by CYP-2D6 or CYP-3A (Bennett and Mohan, 2013). Clinical trials (#NCT02843035) are currently ongoing for Venglustat (Genzyme) to overcome the former SRT compounds’ lack of efficacy for nGD patients. Venglustat can penetrate the blood-brain barrier (BBB) and could specifically be useful in GD3 (Genzyme and a Sanofi Company, 2023).

The use of small chemical chaperones is a promising approach for the treatment of diseases caused by improperly folded proteins like GD in which the majority of the reported disease-causing *GBA1* genetic variants are missense mutations (Jung et al., 2016). They are small molecular weight compounds that are designed to selectively bind to a specific target protein, facilitating its folding and stabilization, as well as promoting lysosomal translocation (Maegawa et al., 2009; Parenti et al., 2015). In the case of lysosome-resident enzymes, genetic mutations can lead to misfolding and subsequent degradation in the proteasome, preventing the enzyme from reaching its intended destination in the lysosome. Chaperone therapy can increase enzyme stability, catalytic activity, and lysosomal translocation, thus restoring normal cellular function (Parenti et al., 2015). However, it is important to note that the presence of the enzyme is required for chaperones to be effective, as these molecules physically bind to the target protein (Muntau et al., 2014). Overall, the development of chaperones represents a promising avenue for the treatment of a range of diseases caused by improperly folded proteins like GD (Liguori et al., 2020). While chaperoning compounds offer hope for stabilizing misfolded proteins in genetic diseases like Gaucher’s, their effectiveness is unfortunately limited by mutation-specificity (Mohamed et al., 2017). The variable efficacy of this compound across different mutations exposes a critical gap in available treatment options for the extensive range of genetic variants in this condition. Therefore, discovering a broader spectrum of chaperone compounds with wider effectiveness across mutations is an urgent priority. Screening chemical libraries consisting of Food and Drug Administration (FDA) and clinically approved compounds has demonstrated practicality and success in identifying small molecules that can function as enzyme enhancement therapy (EET) agents for specific lysosomal enzymes deficient in lysosomal storage disorders. Since ongoing trials for EET or SRT synthesized compounds are still in the early stages of development, and considering the challenges associated with enrolling patients with rare diseases, it is anticipated that market authorization may not be obtained for at least 5–10 years from the present day. In such circumstances, patients with significant nGD features involving the CNS may seek alternative solutions, one of which is repurposing already approved drugs.

In this context, Ambroxol (ABX) has been identified as an EET agent for rescuing misfolded GCase enzymes of different genotypes (Maegawa et al., 2009). In the initial *in vitro* characterization, ABX exhibited a maximal inhibition at a neutral pH (characteristic of the endoplasmic reticulum) and almost undetectable inhibition at the acidic pH found within lysosomes (Maegawa et al., 2009). ABX’s pH-dependent interaction involves binding and stabilizing the misfolded GCase enzyme in the ER to prevent its recognition via the ER quality control machinery. This process facilitates the enzyme’s trafficking to the lysosomes, where ABX dissociates, allowing the mutated GCase enzyme to remain unbound and effectively engage in the breakdown of accumulated substrates (Liguori et al., 2020). Using patient fibroblast cells, treatment with ABX showed promising enzymatic enhancement in various missense variants through direct binding, folding alteration, and the subsequent trafficking correction of the ER-retained ones (Bendikov-Bar et al., 2011; Bendikov-Bar et al., 2013; Kopytova et al., 2021). Modulation and structural analysis showed that ABX interacts with both the enzyme active and allosteric site residues. Based on this discovery, several clinical and pilot studies have been conducted to evaluate the efficacy and safety of ABX as an alternative and/or adjunct therapy in GD patients of different forms (Istaiti et al., 2021; Zhan et al., 2023). Although ABX is well tolerated in most patients, the findings obtained from these studies showed a wide response variability even in cases with similar genotypes (Table 1). Therefore, this review aims to comprehensively explore and summarize all the available clinical findings regarding the use of ABX as a potential therapeutic agent in GD patients of different forms. The focus will be on evaluating the response variability observed in clinical studies to assess the potential of ABX in improving the management of GD.

**TABLE 1:** Summary of the clinical studies performed to evaluate the effectiveness and safety of ABX in Gaucher Disease treatment.

Genotype	Patients number	Phenotype	Adjunct treatments	ABX dosage	Assessment parameters	Improvements	Adverse effects (AEs)	Year and ethnicity	Ref
Different combinations of variants	32	29 GD1	Did not afford treatments	12.7 ± 3.9 mg/kg/day (0.5–6.5 years)	Chitotriosidase activity, glucosylsphingosine level, liver and spleen volumes, and hematologic parameters	More significant among milder and younger cases	Safe (only 3 cases encountered mild and temporary AEs)	2023 China	Zhan et al. (2023)
		1 GD2-3							
		2 GD3							
N370S/N370S N370S/other	40 (16 fully completed the trial)	GD1 (26:ERT, 2:SRT, and 12:Stopped ERT or SRT)	Poor response to ERT or SRT	600 mg/day (12 months)	Platelet counts, lumbar spine T-score, and LysoGb1 levels	- >20% increase in platelet counts and reduction in LysoGb1 - >0.2 improvement in lumbar spine T-score	-no severe AEs −13 patients reported mild AEs	2023 Israel	Istaiti et al. (2023)
R398L/R398L	1	GD2	In combination with ERT	25 mg/kg/day (Initiated at the age of 15 months)	Blood and CSF LysoGb1, neurocognitive and motor development	- Improved visceral and neurological symptoms	no side effects or signs of toxicity	2022 Turkey	Aries et al. (2022)
						−20-fold increase in GCase activity			
V414L/S235P	1	GD1	Never treated	660 mg/day for 2 years	GCase activity and liver assessment	- Significant reduction of hepatic fibrosis	NA	2022 China	Zhang et al. (2022)
						-Enhanced GCase activity			
Different combinations of variants	41	11:GD1 (4 with PD)	In combination with ERT (only 4 were not exposed to ERT)	75–1,485 mg/day (1–76 months)	Demographics, GD type, GD therapy, history of splenectomy, and AEs	25 patients showed enhanced neurological status, physical activity, and reduced fatigue	No severe AEs reported but 12 patients reported mild AEs	2021 Israel	Istaiti et al. (2021)
		27:nGD							
		3:PD							
L444P/H225Q; D409H	2	GD3	Combined with ERT	25 mg/kg/day (Older and younger siblings started at ages 5 years and 7 weeks, respectively)	LysoGb1, GCase activity, CSF CHT activity, neurological assessment	ABX early initiation delayed or halted neurological manifestations prognosis in the younger sibling	No severe AEs reported	2021 Croatia	Ramadža et al. (2021)
L444P/RecNcil	1	nGD	Combined with ERT	30 mg/kg/day (Started at 8 months of age)	LysoGb1, Plasma CHT	Slow progress in the neurological involvement	NA	2020 Taiwan	Chu et al. (2020)
L483P/L483P*	1	GD3	Combined with ERT	7.5 mg/kg/day	Numerical Rating Scale (NRS)	potent analgesic effect with chronic pain that was resistant to standard therapy	No AEs reported	2020 China	Pawlinski et al. (2020)
P1: N188S/IVS2+1G>A	2	nGD	Combined with ERT	25 mg/kg/day	GCase and CHT activities, LysoGb1 levels	P1: LysoGb1 levels, CHT activity, and seizure frequency decreased	No AEs reported	2020 Italy	Ciana et al. (2020)
P2: L444P/L444P						P2: no effects were observed (ABX was started at 60 years)			
L356Wfs*8/R392W	1	GD1	Never exposed to ERT or SRT	10 mg/kg/day increased to 15 mg/kg/day after 6 months for up to 2.5 years	Skeletal imaging assessment, CHT, platelet count, hematological workup	Improved skeletal manifestations as both femoral heads showed significant improvement in sphericity and reshaping	No AEs reported	2019 China	Jiang et al. (2020)
N227S/R296Q	4	nGD	Combined with ERT for 4.5 years	1.5–27 mg/kg/day	GCase activity, biochemical, safety, and neurocognitive findings	Seizure frequencies markedly decreased; neurocognitive function was improved, and LysoGb1 levels were normalized	High-dose ABX was well-tolerated with no severe AEs	2019 Korea	Kim et al. (2020)
N227S/V211Ffs									
F252I/L483P*									
G377S/G195E	2	GD3	Combined with ERT	25 mg/Kg/day	LysoGb1 levels and neurological status	Improvement in ambulation, and LysoGb1 levels	No AEs reported	2019 Canada	Charkhand et al. (2019)
N188S/R463H						Lower seizure duration in one patient only			
N188S/G193W	5	nGD	Combined with ERT	25 mg/kg/day	GCase activity, LysoGb1 levels, Neurological assessment	Myoclonus, seizures, and pupillary light reflex dysfunction markedly improved in all patients and two were able to walk again	No serious AEs reported	2015 Japan	Narita et al. (2016)
N188S/?									
F213I*/RecNciI									
D409H/IVS10‐1									
N370S/N370S N370S/84GG	12	GD1	Unable or unwilling to receive ERT	2 capsules of 75 mg/day (6–12 months)	Liver and spleen volumes, platelet count, hemoglobin, and CHT activity	ABX stabilized and enhanced the clinical status of GD1 patients not receiving ERT.	Two patients dropped out due to hypersensitivity reactions. No other AEs	2013 Israel	Zimran et al. (2013)

*L483P, F252I, and N409S are alternate nomenclatures for L444P, F213I, and N409S respectively due to the use of different *GBA1* transcripts.

## 2 Ambroxol as a potential pharmacological chaperone therapy in GD

ABX is an oral mucolytic drug available over the counter since many years as a cough medicine in various countries with the exception of the United States (Cazan et al., 2018). It has been demonstrated to be highly effective as an expectorant, anti-inflammatory, and antioxidant agent due to its extensive history of human use in treating airway mucus hypersecretion diseases such as chronic bronchitis, asthma, and cystic fibrosis (Ollier et al., 2020). Moreover, ABX has been employed as an alternative preventive therapy for hyaline membrane disease in newborns by enhancing the secretion of surfactant in the lungs (Ollier et al., 2020). ABX showed an anesthetic effect in several studies which is mainly due to its inhibitory effects towards Na^+^ channels, Ca^2+^ channels, and glutamate receptors (AMPA type) effectively suppressing symptoms of chronic, inflammatory, and neuropathic pain *in vivo* (Jarvis et al., 2007; Weiser, 2008). Unlike other blockers, ABX CNS‐related toxicity and adverse effects were mainly reported with very high doses (Istaiti et al., 2021). ABX is available in multiple prescription formulations. ABX immediate-release oral form (tablets, soft pastilles, granules, syrup, and oral solution) with a half-life of ≈10 h which is prescribed twice per day (Cazan et al., 2018). The ABX extended-release formulations on the other hand have a longer half-life that maintains the drug levels in the blood over 24 h reducing the frequency of administration to once per day. Beyond its chaperone activity, previous data indicate that ABX possesses additional anti-inflammatory and antioxidant properties (Bianchi et al., 1990; Kantar et al., 2020). This suggests broader therapeutic potential for ABX in lysosomal storage diseases (LSDs), including GD, by targeting additional aspects of their pathogenesis.

To establish the potential use of ABX as an EET for GD treatment, several studies have been conducted. Using patients’ derived cells, cellular assessment of ABX’s chaperoning activity demonstrated positive enhancement in mutated GCase expression, residual activity, and/or glucosylceramide accumulation for different GD disease-causing variants, including N370S, L444P, N227S, F213I, G232W, R159W, and G241R (Maegawa et al., 2009; Bendikov-Bar et al., 2011; Narita et al., 2016; Kim et al., 2020). In GD3 fibroblast cells with L444P (the second most prevalent following F213I) variant, ABX helps to release ER-retained enzymes to lysosomes, resulting in a noticeable increase in residual activity (Ivanova et al., 2021). Notably, the observed impact was not universally present in cells derived from L444P homozygous patients, with some displaying no discernible positive response (Bendikov-Bar et al., 2011). Cells from GD1 patients carrying L444P genotypes in a compound heterozygous state with another *GBA1* variant exhibited a more significant overall improvement compared to L444P homozygous forms (Kopytova et al., 2021). This indicates that there is a certain level of response variability towards ABX even with the same genotype. Protein modeling studies have shown that ABX interacts with both active and non-active site residues of the GCase enzyme, which is consistent with its mixed-type response in different cells (Maegawa et al., 2009). ABX’s mechanism of restoring GCase levels and function may involve direct binding for enzyme stabilization, upregulation of expression through the activation of the transcription factor Nrf2, changes in lysosomal pH to enhance enzymatic activity, and induction of GCase expression through TFEB-mediated lysosomal regulation (Sardiello, 2016; Kopytova et al., 2021). Additionally, ABX significantly enhances both trafficking and activity of ER-retained GCase in patients’ fibroblast cells for various GD variants and disease forms. It is noteworthy that the level of improvement varies among the variants; hence, the chaperoning effect of ABX is variant-dependent (Bendikov-Bar et al., 2013; Luan et al., 2013; Suzuki et al., 2013). Therefore, clearing the ER compartment from the retained mutated GCase through ABX ameliorates the ongoing ER stress resulting from the underlying accumulation. Data from different studies have shown that ABX has the potential to promote proper protein folding, reduce the accumulation of misfolded proteins, and enhance the ER-associated degradation (ERAD) pathway (Remondelli and Renna, 2017). It also plays a significant role in ER stress relieve as evident from Unfolded Protein Response (UPR) markers reduction in the 18-day-old ABX-treated GD-drosophila model (Cabasso et al., 2019). The treatment significantly lowers the dihydroethidium marker of oxidative stress in cultured fibroblast cells carrying various *GBA1* mutations (homozygous and heterozygous) (McNeill et al., 2014). In normal mice, ABX showed elevated GCase activity in various tissues, including the spleen, heart, and brain, without causing any severe adverse effects indicating its wide range of tissue bioavailability (Luan et al., 2013). ABX’s capability of crossing the BBB was further supported in healthy nonhuman primates (Migdalska-Richards et al., 2017). Besides its chaperoning effect, ABX also affects lysosomal biogenesis, autophagy, secretory pathways, and mitochondrial function (Ambrosi et al., 2015; Fois et al., 2015; Magalhaes et al., 2018; Ivanova et al., 2021; Kopytova et al., 2021). ABX has been discovered to boost lysosomal activity and facilitate the removal of protein aggregates in different neurological-related disorders including GD (Bonam et al., 2019). Autophagy was found to respond differently to ABX treatment as it influences the redirection of the accumulated cellular cargo toward the secretory pathway (exocytosis) by blocking autophagy in primary cortical *GBA1* ^*−/−*^ mouse neurons (Magalhaes et al., 2018). On the other hand, autophagy was activated in human iPSC differentiated neurons obtained from PD cells carrying *GBA1* mutation through elevated LC3-II and p62 expression upon ABX treatment (Yang et al., 2017). ABX was shown to target lysosomal dysfunctions in human *GBA1* mutated fibroblasts through Cathepsin D, LIMP2, and Sap C modulation, facilitating GCase folding and proper trafficking (Ambrosi et al., 2015). ABX facilitates lysosomal-autophagosomal fusion, enhancing protein degradation and potentially reducing ER stress and lysosomal dysfunction for improved protein folding and clearance (Magalhaes et al., 2018). Additionally, ABX induces mitochondrial activation, evidenced by an augmentation in both mitochondrial mass and density, along with the activation of mitochondrial membrane potential as demonstrated in GD2-3 fibroblast cells (Ivanova et al., 2021). Mitochondrial content and lysosomal biogenesis are also enhanced by ABX through peroxisome proliferator-activated receptor gamma coactivator 1-alpha (PGC1-α) and TFEB, respectively (Magalhaes et al., 2018). The compound has a significant potential in the treatment of PD and neuronopathic GD by reducing both α‐synuclein and phosphorylated α‐synuclein levels in vivo transgenic murine models (Migdalska-Richards et al., 2016).

Given ABX’s over-the-counter availability and oral formulation, its effectiveness for certain GD manifestations could offer significant benefits, particularly for patients encountering challenges with their current treatment regimens, especially those with neurological symptoms. Although they have a lower prevalence, the nGD subtypes are more prevalent in developing countries of the middle east and Ascia-Pacfic (Al-Jasmi et al., 2013; Roshan Lal and Sidransky, 2017; Fateen and Abdallah, 2019; Castillon et al., 2022). Providing a cheaper therapeutic option like ABX can solve the limited availability of ERT and SRT expensive medications to this group of patients. As the doses needed to maintain the pharmacological chaperone (PC) effect of ABX are significantly higher compared to over-the-counter doses (the highest: 75 mg/tablet) available in the market, ABX formulations are limited to lower concentrations. This may result in the need for consuming multiple capsules or a large amount of syrup daily, in adults and children, respectively (Istaiti et al., 2021). Further research and clinical studies investigating the chaperoning effect of ABX in treating GD can help encourage pharmaceutical companies to develop appropriate formulations and dosage regimens for the drug in the market. Unfortunately, the exclusive patent (European Patent Office Register Application number: EP3145491) for this drug was granted to a small company that lacks the financial resources to conduct a clinical trial for its potential use as a treatment for GD (European Patent Register, 2023). Furthermore, larger pharmaceutical companies have not expressed interest in this drug, partly due to the abundance of generic versions available for purchase online (Istaiti et al., 2021).

## 3 Clinical studies on Ambroxol

### 3.1 Gaucher disease assessment parameters and monitoring

To date, there is no conclusive biomarker that can reliably predict the risk associated with all aspects of GD morbidity. Although several potential biomarkers have been recognized, each comes with inherent limitations, making clinical parameters the primary method for establishing treatment objectives and evaluating outcomes. Clinicians must rely on their expertise to determine the most effective monitoring protocol that is tailored to each case based on the GD form presented and the treatment type. Over the duration of any therapeutic intervention, patients undergo standard clinical monitoring that includes regular clinical status evaluation, radiological evaluations, hemogram, enzymatic activity, and different disease biomarkers (Stirnemann et al., 2017).

Since ABX binds directly to the mutated GCase enzyme, assessing the enzymatic activity is a key indicator to determine the effectiveness of the ABX treatment and whether the mutated enzyme function is improving during the treatment period (Han et al., 2020). This information can help clinicians adjust the drug dosage based on the rate of increase in residual enzymatic measurement. The test can be performed regularly using a fresh peripheral blood leukocytes sample (Hurvitz et al., 2019; Dardis et al., 2022). As previously mentioned above, ABX additionally modifies the enzyme conformation through an allosteric site interaction which its enhancement effect might not be detected directly through enzymatic activity measurement (Maegawa et al., 2009). Therefore, other biomarkers like substrate accumulation parameters can be measured. While the impairment of GCase enzyme in GD patients results in the accumulation of its GlcCer substrate, several additional lipids like glucosylsphingosine (LysoGb1, derived by ceramidase deacylation of GlcCer) are enriched in GD patient samples (Dekker et al., 2011). The quantification of LysoGb1 through liquid chromatography-mass spectrometry in patient plasma, dried blood, and cerebrospinal fluid (CSF) samples represents the most sensitive approach. This analytical method is highly effective, and accurate that has the potential to provide valuable insights into GD therapeutic monitoring including ABX treatment (Hurvitz et al., 2019). To obtain a comprehensive understanding of a patient’s clinical progress, it is essential to consider multiple other biomarkers in the clinical assessment. Studies have shown that the enzyme chitotriosidase (CHT) and the chemokine CCL1811 are produced by Gaucher cells and released into the patient’s circulation (Hollak et al., 1994). These two proteins are potential biomarkers because their plasma levels are significantly elevated in GD patients, and change as the disease progresses as well as after a treatment intervention like ABX (Zhan et al., 2023).

Platelet count monitoring is crucial for GD management and treatment evaluation as more than 90% of affected patients suffer from thrombocytopenia due to bone marrow infiltration and splenic sequestration (Hollak et al., 2012). Although it is less common, anemia and hemoglobin level measurement in 50% of the cases can help in assessing the efficacy of the ongoing treatment (Charrow et al., 2000). Abdominal magnetic resonance imaging (MRI) is used to measure the dimensions of the liver and spleen, to evaluate their volume and morphology to assess the progression of hepatosplenomegaly. The decrease in organ volumes as a result of treatments is usually a slow process and should be monitored as a long-term effect (Elstein et al., 2011). Improvement of bone abnormalities is best followed through radiology via bone MRI to check for bone marrow infiltration (Bone Marrow Burden score), bone infarction, avascular necrosis, and osteoarthritis (Vom Dahl et al., 2006). Bone densitometry is measured via X-ray imaging to diagnose and monitor osteopenia/osteoporosis in adults and older children affected with GD. Patients with nGD forms require additional neurological monitoring. The combination of electroencephalogram (EEG) recording and brain MRI is regularly performed to evaluate the improvement or progression speed of nGD-associated CNS symptoms along any therapeutic intervention. The tests mainly check for brainstem-evoked potential, swallowing studies, and neuro-ophthalmologic evaluation should be done at regular intervals (Stone et al., 2023). The frequency of clinical examination is mostly decided by the patient’s clinician, but it is advisable to run full laboratory tests and imaging when required every 6 months for pediatric GD patients as a function of disease progression and drug monitoring.

### 3.2 Ambroxol effectiveness and response variability in Gaucher disease patients

Ever since its discovery as a chaperone in 2009, ABX repurposing efficacy in the treatment of GD patients of different forms has been evaluated in a total of 14 clinical trials and studies summarized in Table 1 (Maegawa et al., 2009). The positive responses reported in these published data have encouraged physicians and patients to consider the off-label use of ABX for GD in combination with the currently available ERT/SRT treatments or as an alternative when such solutions fail to induce any clinical improvement. Also, it has been an option when ERT or SRT is not available or affordable for some patients. The majority of the trials conducted on ABX have reported it to be safe and well-tolerated in most cases even during long-term treatments. However, understanding the reported adverse effects in detail is key to minimizing their impact and preventing future occurrences. Among the 14 reported trials, 4 extensive primary studies explored ABX’s impact on a large cohort of GD patients (Zimran et al., 2013; Istaiti et al., 2021; Istaiti et al., 2023; Zhan et al., 2023). Notably, 3 of these studies took place in Israel, reflecting its higher GD prevalence compared to other populations (Zimran et al., 2013; Istaiti et al., 2021; Istaiti et al., 2023).

In an observational data involving a cohort of 28 GD patients representing the three clinical forms who completed the proposed treatment, ABX (administrated over 2.6 ± 1.7 years) successfully improved the overall clinical status in most of the enrolled patients including reduced fatigue, more energy, fewer nosebleeds, and disappearance of petechia on the skin (Zhan et al., 2023). Patients with nGD experienced stable or improved neurological manifestations during the treatment. Hematological levels were enhanced within the first year of the treatment at which 76.2% and 23.8% of the participants met the hemoglobin and platelet count goals, respectively. Improvements in platelet count were the hardest to achieve mainly due to the associated spleen enlargement. Among the eight patients who were assessed for hepatosplenomegaly, seven patients achieved the spleen volume goal, and six reached the desired liver volume, resulting in an average reduction rate of 29.5% and 21.1% respectively. A decrease in the primary GD biomarkers (LysoGb1 and CHT) levels was observed and consistently maintained throughout the treatment period in 76.9% of the patients with elevated LysoGb1 (26 patients) and 93.8% of those with elevated CHT (16 patients). Eleven patients of the cohort who underwent a prolonged (>4.5 years) ABX treatment exhibited more substantial reductions in CHT (77.8%) and LysoGb1 levels (52.8%). A strong response to ABX treatment was observed in splenectomized patients who showed decreased LysoGb1 and CHT levels ranging from 16.9% to 77.9% and 24.2%–55.4%, respectively (Zhan et al., 2023). ABX may require an extended duration to reverse the pathological glycosphingolipids accumulation which was observed in two patients who had a stable clinical course with no significant improvement detected during the early stages of the treatment, but after the continuous administration for 4 years eventually led to a significant reduction in LysoGb1 and CHT levels.

In parallel to the previously mentioned trial, a prospective open-label clinical trial (investigator-initiated research) was conducted to assess the application of high-dose ABX in treating two subgroups of GD1 patients; those who exhibited no response to ERT and/or ERT, and patients who had never been exposed to any of the available therapies (Istaiti et al., 2023). Out of the forty patients enrolled, sixteen completed the study of which ten were on ERT, one on SRT, and five never had a treatment adjunct to the given ABX protocol. The study proposed the partial potential of high ABX dosage in improving platelet count (5/16), LysoGb1 (3/16), and lumbar spine T-score (measurement of bone densitometry) (6/16) with a success rate ranging from ∼20% to 40% encouraging further studies (Istaiti et al., 2023). Notably, four patients experienced a notable decrease in LysoGb1 levels after 1 month of ABX treatment, but unfortunately, these levels subsequently increased over time failing to meet the desired outcome by the end of the study. The same group conducted an earlier observational study from a cohort of 41 GD patients (GD1: 11, nGD:27) exposed to off-label ABX (Istaiti et al., 2021). Patient data were collected from 13 different centers to establish an investigator-initiated registry II-Reg, which was registered on ClinicalTrial.gov (ID: NCT04388969) (Istaiti et al., 2021). Although, 12 patients reported mild adverse effects, clinical benefits of the underlying ABX (75–1,485 mg/day) treatment were observed in 25 patients, leading to stable or improved neurological status, enhanced physical activity, and reduced fatigue. Among the GD1 group, 6 out of 11 patients showed overall wellbeing improvement, with some exhibiting enhanced LysoGb1 levels (an 85% reduction in one patient) and increased platelet counts. In contrast, patients with nGD demonstrated a more favorable response (20/27) towards ABX treatment with 16 patients experiencing stabilized or improved neurological status, along with improvements in physical activity, LysoGb1 levels, and reduced pain (3 out of 27). The seven patients who did not show improvement mainly discontinued ABX due to adverse effects or medication reimbursement issues, with one patient, unfortunately, passing away due to deteriorating clinical status. In summary, this study underscores the potential of ABX in improving the clinical manifestations of GD patients when used alongside their existing ERT medications.

Shaare Zedek Medical Center’s Gaucher Unit tested ABX as a possible EET treatment in the first clinical trial with a cohort of GD1 patients who had never been on ERT (Zimran et al., 2013). They received an off-label ABX dosage of 150 mg/day for 6 months. During the study, one patient demonstrated a significant improvement in hemoglobin levels and platelet counts, with increases of 16.2% and 32.9%, respectively. His spleen and liver volumes decreased by 2.9% and 14.4%. Alongside this patient, two others continued ABX treatment for an additional 6 months. During this period, all three patients experienced ongoing reductions in spleen volume, ranging from 6.4% to 23.4%. Their hemoglobin levels and liver volumes remained stable, while the patient who initially responded well recorded a further increase in platelet counts by 52.6%. The substantial clinical impact on this patient initiated subsequent investigations, with a specific focus on potential adjustments to the ABX dosage, particularly considering the responsive patient’s low BMI (17.1) compared to the other participants.

Like CNS-related manifestations, improvement of skeletal symptoms and complications is limited with the current approved GD therapies due to the inability of ERT enzymes to reach affected tissues in optimal concentrations (Jiang et al., 2020). A clinical case study of a GD1 patient who underwent a gradual ABX dose escalation revealed a significant therapeutic impact on the bilateral aseptic necrotic lesions linked to his presented phenotype (Jiang et al., 2020). The 5-year-old patient was suffering from severe pain in the bilateral lower extremities, restraining her from walking independently due to the aseptic necrosis detected in the bilateral femoral head of her hip. As neither ERT nor SRT was applicable, she was enrolled in a clinical protocol under compassionate use, receiving an initial 10 mg/kg/day dosage of ABX for 6 months to assess her tolerance, followed by an extended treatment period of 2 years at a dosage of 15 mg/kg/day. Three years post-treatment, the patient maintained a remarkable recovery of the femoral head sphericity and reshaping that is primarily attributed to the prevention of further progression of ischemic lesions in the femoral head’s epiphysis, which has contributed to the maintenance of remodeling and nearly normal growth in the femoral heads.

Clinical data suggests that ABX when added to standard therapy, can effectively manage pain associated with GD. This allows patients to rely less on traditional pain medications and improves their quality of life. ABX has shown analgesic properties by inhibiting calcium channels, which reduces the transmission of sensory signals from primary afferent neurons to the dorsal spinal cord. This mode of action was found to relieve the pain in a 38-year-old GD3 female patient who suffered from chronic soreness around the lumbar part of her spine and pelvis, which worsened during more intensive physical activity (Pawlinski et al., 2020). Despite undergoing various painkiller treatments, the pain persisted until ABX was incorporated into her treatment plan. Initially, she was given a dose of 150 mg/day, which was gradually increased to 450 mg/day. Over several months, her need for painkillers significantly decreased, and her pain levels became more manageable at which the pain intensity decreased from NRS 7 to below 3. Subsequent attempts to reduce the ABX dosage resulted in the return of the pain.

The last two studies referenced above emphasize ABX’s potential to cross the blood-brain barrier and access hard-to-reach tissues, such as bone. This was initially administered to patients who were unresponsive to ERT/SRT and presented with CNS-related GD symptoms. Administering ABX, particularly at high dosages, to individuals with nGD (GD2 and GD3) has been shown to significantly improve their condition (Narita et al., 2016; Charkhand et al., 2019; Chu et al., 2020; Ciana et al., 2020; Kim et al., 2020; Pawlinski et al., 2020; Ramadža et al., 2021). It exerts a neuroprotective impact by slowing down the progression of the affected tissue in the brain by reducing the build-up of the accumulated substrates which is evident in decreased levels of LysoGb1 biomarker in patients’ both plasma and CSF samples. As a result, nGD patients have reported improvements in seizure frequency and duration, motor function, cognitive abilities, and quality of life at various degrees following ABX treatment. Myoclonus in affected patients showed progressive improvements with increasing doses of ABX where they were able to regain their ability to stand steadily, maintain balance, and either walk again or experience improved ambulation (Narita et al., 2016; Charkhand et al., 2019). The most favorable improvements were witnessed in younger patients even with known responsive genotypes like L444P (Ciana et al., 2020). The patient was in his 60s when he started taking ABX and showed no phenotypic enhancement of any level even with increasing the drug dosage. Early initiation of ABX treatment at a younger age showed more substantial improvements by postponing or averting neurological progression in nGD patients (Ramadža et al., 2021). In Ramadža et al. report, two GD3 siblings were administered a combination of ABX and ERT medication at varying stages of their disease (Ramadža et al., 2021). The older sibling, diagnosed at 4.5 years with severe neurological symptoms, experienced substantial improvement and stability with ABX treatment. In contrast, the younger sibling, who started treatment at just 7 weeks old, exhibited cognitive challenges without the development of other neurological symptoms his brother suffered from. This suggests that there may be a threshold in the progression of GD beyond which ABX therapy might not be effective, particularly in cases of advanced disease stages, therefore, initiating ABX treatment early in the disease course could be critical for its effectiveness.

### 3.3 The significance of Ambroxol dosage and its role as a supplementary treatment

A major limitation in these clinical trials is the selection of the proper ABX dosage. The choice of the amount of the drug given to patients might seem somewhat random, and the selected dosage in the reported data may be either too much or too little. The clinical data presented in this review article suggests that ABX has a greater chaperoning effect in GD patients when used at high dosages rather than over-the-counter use. In the first pilot study conducted on GD1 patients, where ERT was not an option, the dosage administered was kept low due to safety concerns. However, it is possible that the dosage was too low as patients with low BMI showed better measurable improvements and the thinnest patient (BMI = 17.1) among the recruited cohort had a robust response in all disease parameters tested (Zimran et al., 2013).

In Istaiti et al. study (2023), most patients who were exposed to lower doses did not show clear benefits except for a few who had low LysoGb1 biomarkers from the start before drug administration (Istaiti et al., 2023). This suggests that the given 600 mg/day dose might be necessary for most affected patients included in the study, even though some studies in the past hinted at the use of lower doses. Unlike data obtained from nGD trials, the authors suggested that higher doses are probably unnecessary in GD1 patients as there is no need to cross BBB (Narita et al., 2016; Istaiti et al., 2021). To reach a responsive high ABX dosage, patients will need to take a lot of capsules daily (8 per day for the 600 mg/day) which is another limiting factor and cause for dropout in some studies. As previously mentioned above, ABX was given as a pain relief medication at 450 mg/day over several months which was effective in reducing the underlying chronic pain but the subsequent attempts to reduce the ABX dosage resulted in the return of the pain (Pawlinski et al., 2020).

ABX has been initially identified to target patients who did not benefit from the current therapeutic protocols or those who suffer from neurological manifestations that do not respond well to such treatments. However, ABX also has been investigated as a potential complementary therapy for GD patients, particularly those with neurological involvement. These patients often receive standard treatments like ERT or SRT alongside ABX, as part of clinical trials or under compassionate use protocols. In a 2016 pilot study, five patients with GD were included, four of whom were diagnosed with an nGD form and received high-dose ABX treatment in combination with ERT. Apart from its tolerability and wide distribution into affected tissues including the brain, the results showed significant response as concluded from the enhanced GCase activity, LysoGb1 levels, and overall physical and neurological wellbeing of the patients (Narita et al., 2016). As indicated in previous studies, a notable reduction in LysoGb1 levels was reported among GD1 patients who received ERT or SRT treatments (Zhan et al., 2023). In patients who completed 209 weeks of Velaglucerase alfa treatment, the median substrate accumulation reduction exhibited an impressive 85.7% reduction. Similarly, for GD1 patients undergoing eliglustat therapy for 8 years, LysoGb1 levels decreased by approximately 60% from baseline after just 1 year followed by a remarkable 92% reduction after 8 years of treatment indicating that better responses in ABX-treated patients are associated with long-term ERT. These findings highlight the potential of ABX to provide supplementary benefits in further improving the overall management of GD.

### 3.4 Preclinical assessment

Variants such as R392W, F76V, N227S, R87W, M75V, R159W, L188F, L303I, V414L, and R502C have previously been associated with a late-onset and milder GD phenotypes. Patients harboring either two mild variants or one in association with another variant (L444P, RecNcil, R535C, R202X, N227S, N227K, and V230G) linked to more severe phenotype responded well to ABX compared to those patients carrying two severe variants (Zhan et al., 2023). Given that, it has been observed that the response to the ABX chaperoning effect is not only specific to mutations but also varies among patients. Even patients with the same genotype may respond differently to ABX treatment, requiring dosage optimization or displaying varying degrees of phenotypical enhancement. To address this issue, preclinical assessment has been suggested, wherein ABX treatment can be tested and optimized using the patient’s cells, such as fibroblast cells and peripheral blood mononuclear cells (PBMC) derived cells, *in vitro* (Bendikov-Bar et al., 2011; Kopytova et al., 2021). The L444P missense variant variable response to ABX is the best example to support the stated point. The mutation is the second most frequent mutation identified in nGD patients and has been reported in GD1 patients when inherited in a heterozygous state (Ciana et al., 2020).

Although the initial *in vitro* evaluation of ABX’s effect on L444P genotype demonstrated no positive enhancement, a significant increase in GCase activity was observed in patient’s cells harboring the compound heterozygous genotype of L444P (Maegawa et al., 2009). Despite that, computational and docking studies have demonstrated a strong binding affinity of ABX towards L444P mutated GCase (Thirumal Kumar et al., 2019). In animal studies, oral administration of 4 mM ABX for 12 days showed a significant increase in the GCase activity in different brain tissues of L444P/+ transgenic mice (Migdalska-Richards et al., 2016). On the other hand, the data at the clinical level was highly varied showing contradictory results for this variant. In a 2021 clinical investigation of ABX effect on 41 GD patients carrying different GD variants of which 10 with the homozygous state of L444P and 7 were in a compound heterozygous state with other variants (Istaiti et al., 2021). Among the L444P homozygous patients, ABX was able to enhance the neurological status of two patients and arrest the neurological progression in five other patients without any noticeable improvements. It also managed to improve the overall physical status of another two patients and decrease their complaints of underlying pain. Of the seven patients with the compound heterozygous variants with L444P, three showed improvements in their neurological status while two patients did not show any further deterioration. It is worth noting that the patient carrying the null GD variant (L444P/IVS2+1) showed significant neurological enhancement also (Istaiti et al., 2021). In Croatian GD3-affected siblings (L444P/H225Q; D409H), ABX showed rapid improvement in neurological manifestations and later stabilized the older sibling’s clinical condition (Ramadža et al., 2021). However, the younger sibling, who started the ABX protocol at 7 weeks of age, did not exhibit any severe neurological symptoms indicating that ABX delayed or even halted the evolution of neurological manifestations regardless of the underlying genotype. Also, both *in vitro* (using his fibroblast cells) and clinical investigations of ABX therapy in an Italian 60-year-old nGD patient carrying the L444P/L444P genotype showed no effect (Ciana et al., 2020). The patient was undertaking ERT medication but due to the worsening of the neurological involvement he began on high ABX dosage for 4 months with no success. This is another case that may support the importance of the early administration of ABX as an EET medication in GD especially with patients presented with neurological crises with irreversible consequences. It appears that factors other than the patient’s genotype could affect their response to ABX. One such factor is the patient’s age, especially if they are experiencing severe neurological or skeletal crises that EET cannot reverse. Additionally, polymorphisms or genetic modifiers may have some influence as well. Since L444P affects the folding and trafficking of the mutated GCase, individual differences in the ER quality control machinery components involved in proteostasis likely play a major role in this process (Bendikov-Bar et al., 2011). Therefore, it is highly recommended to test the response to ABX *in vitro* before initiating chaperone therapy, even in patients with responsive variants.

Chu et al. clinical report has also demonstrated that the *in vitro* ABX pre-assessment may not apply to all patients (Chu et al., 2020). Although it was previously reported as an unfavorable variant via *in vitro* analysis, an nGD infant carrying L444P/RecNcil variant followed a high ABX dosage started at the age of 6 months showed significant clinical enhancements with no further regression. The obtained outcome might be a result of the early initiation of the therapeutic intervention (ERT and ABX) when the patient’s condition was less severe compared to progressive cases reported in older patients carrying the same genotype and did not benefit from ABX.

### 3.5 Safety and adverse effects

ABX treatment has generally been considered safe in most of the reported clinical studies, with limited adverse effects or severe cutaneous adverse reactions (SCARs). Most patients tolerate ABX well, and it is often used as an over-the-counter mucolytic agent (Cazan et al., 2018). Adverse effects are typically mild and transient, including issues like gastrointestinal discomfort with symptoms like nausea, vomiting, or stomach upset which mostly tend to resolve on their own or with dose adjustments (Zhou et al., 2022). However, the safety and adverse effects may vary among individuals and require careful monitoring, especially with long-term administration and high dosages (>120 mg/day). ABX dosage has been thoroughly evaluated in preclinical studies involving patients with diverse medical conditions to determine its safety profile. These studies have yielded promising results, highlighting the potential of high-dosage ABX in managing various medical conditions. For example, a study involving pregnant women experiencing premature labor or premature rupture of membranes showed that intravenous administration of 1,000 mg of ABX for 4 hours daily over 3 days significantly reduced the incidence of infant respiratory distress syndrome (IRDS) and perinatal morbidity and mortality, without any adverse effects (Laoag-Fernandez et al., 2000). In two distinct studies, ABX has exhibited its capacity as an antiallergic agent and a local anesthetic, and notably, no toxicity was reported in either case (Fischer et al., 2002; Gibbs, 2009). Similarly, intravenous administration of high-dose ABX at 990 mg/day for five consecutive days after cervical spinal cord injury surgery has been shown to enhance oxygenation and prevent postoperative respiratory complications with limited adverse effects (Li et al., 2012).

In the context of GD, out of all the ABX-treated patients followed in the clinical trials summarized in Table 1, only two were reported to develop hypersensitivity reactions and discontinued the treatment (Zimran et al., 2013). It is worth noting that these two patients were not eligible for ERT treatment due to similar reactions and the developed hypersensitivity is not only developed due to ABX treatment. In the latest clinical trial involving a high ABX dosage (12.7 ± 3.9 mg/kg/day) administered over a duration spanning from 6 months to 6.5 years to 28 GD patients representing all three forms of GD, only a minor subset of three patients encountered mild and temporary adverse events, including nausea, salivation, diarrhea, and rash providing additional evidence of the long-term safety of high ABX dosage (Zhan et al., 2023). Despite its reported excellent safety profile, 13 ABX-treated patients (600 mg/day) dropped out of a clinical study involving 40 candidates due to the development of mild adverse events associated with the treatment (Istaiti et al., 2023). During the treatment, there were some reported side effects such as cough, nausea, abdominal pain, diarrhea, and headache. However, none of these side effects were severe and they were mostly resolved once the treatment was discontinued. One patient had a temporary asymptomatic rise of the liver enzymes, but it rapidly resolved upon the termination of the ABX treatment. Additionally, eight cases of hypouricemia were observed, but they were transient and resolved on their own. While twelve patients out of the initial 41 participants encountered similar mild adverse events, only eight dropped out (two due to ABX-unrelated death) from the study which was primarily established to initiate a registry of ABX treatment in GD in 2021 (Istaiti et al., 2021). The other conducted trials did not report any further severe adverse effects with the administered dosages of ABX (Table. 1). However, like any medication, individual responses may vary, and careful monitoring is essential. Further research and long-term studies are needed to comprehensively assess the safety and potential adverse effects of ABX, especially in various clinical contexts and patient populations.

## 4 Conclusion

An invited commentary article was recently published, in which the authors examined the use of ABX treatment in GD (Weinreb and Goker-Alpan, 2023). They noted that while the treatment may be regarded as ambitious, it is also associated with a sense of ambivalence, owing to the lingering uncertainty surrounding several critical clinical issues. The unresolved nature of these issues makes it difficult to fully endorse the use of ABX in the treatment of GD. Concerns lie around potential safety issues, the lack of official data, and the absence of standardized treatment protocols. Despite its potential therapeutic effect seen in many cases, the lack of ABX pharmacokinetic data to adjust the appropriate responsive dosage limits the ability of clinicians to prescribe GD patient-specific prescription (dose and formula). Beyond the associated *GBA1* genotype, patients response to ABX may be influenced by other factors like different genetic modifiers, epigenetics, and drug metabolism (Davidson et al., 2018). In addition, major GD registries (International Collaborative Gaucher Group and Gaucher Outcome Survay), holding thousands of patient records and data, have not recognized ABX as a valid treatment, further contributing to the ongoing hesitation (Istaiti et al., 2021). To bridge this gap and facilitate data collection, collaborative efforts between clinical research entities and the International Gaucher Alliance are needed to support clinical studies like the ones mentioned in this review article that involve the recruitment of more patients of all representations (ethnicities, genotypes, clinical phenotypes, and treatment options).
